# Crop Management Impacts the Soybean (*Glycine max*) Microbiome

**DOI:** 10.3389/fmicb.2020.01116

**Published:** 2020-06-03

**Authors:** Reid Longley, Zachary A. Noel, Gian Maria Niccolò Benucci, Martin I. Chilvers, Frances Trail, Gregory Bonito

**Affiliations:** ^1^Department of Microbiology and Molecular Genetics, Michigan State University, East Lansing, MI, United States; ^2^Department of Plant, Soil and Microbial Sciences, Michigan State University, East Lansing, MI, United States; ^3^Genetics and Genomic Sciences Program, Michigan State University, East Lansing, MI, United States; ^4^Department of Plant Biology, Michigan State University, East Lansing, MI, United States

**Keywords:** soybean, agricultural management, rDNA, amplicon sequencing, plant-microbe interactions

## Abstract

Soybean (*Glycine max*) is an important leguminous crop that is grown throughout the United States and around the world. In 2016, soybean was valued at $41 billion USD in the United States alone. Increasingly, soybean farmers are adopting alternative management strategies to improve the sustainability and profitability of their crop. Various benefits have been demonstrated for alternative management systems, but their effects on soybean-associated microbial communities are not well-understood. In order to better understand the impact of crop management systems on the soybean-associated microbiome, we employed DNA amplicon sequencing of the Internal Transcribed Spacer (ITS) region and 16S rRNA genes to analyze fungal and prokaryotic communities associated with soil, roots, stems, and leaves. Soybean plants were sampled from replicated fields under long-term conventional, no-till, and organic management systems at three time points throughout the growing season. Results indicated that sample origin was the main driver of beta diversity in soybean-associated microbial communities, but management regime and plant growth stage were also significant factors. Similarly, differences in alpha diversity are driven by compartment and sample origin. Overall, the organic management system had lower fungal and bacterial Shannon diversity. In prokaryotic communities, aboveground tissues were dominated by *Sphingomonas* and *Methylobacterium* while belowground samples were dominated by *Bradyrhizobium* and *Sphingomonas.* Aboveground fungal communities were dominated by *Davidiella* across all management systems, while belowground samples were dominated by *Fusarium* and *Mortierella*. Specific taxa including potential plant beneficials such as *Mortierella* were indicator species of the conventional and organic management systems. No-till management increased the abundance of groups known to contain plant beneficial organisms such as *Bradyrhizobium* and Glomeromycotina. Network analyses show different highly connected hub taxa were present in each management system. Overall, this research demonstrates how specific long-term cropping management systems alter microbial communities and how those communities change throughout the growth of soybean.

## Introduction

Soybean (*Glycine max* L.) is the third most valuable plant crop worldwide with important uses in feed, as an oilseed crop, and as a nutritional source (Food and Agriculture Organization of the United Nations, 2012). Alternative cropping strategies are becoming increasingly common in row crop agriculture in order to manage resource inputs and soil health (Claassen et al., 2018). For example, the use of no-till and reduced tillage strategies have increased in row crops since the early 2000’s in the United States (Claassen et al., 2018). Reduced tillage strategies are especially prevalent in soybean, representing 70% of planted acreage in 2012 (Claassen et al., 2018). In addition to time and fuel-cost savings, no-till farming deposits organic carbon closer to the surface of the soil, which acts as an organic mulch and may lead to improved crop growth and health (Pan et al., 2009; Powlson et al., 2014). In wet conditions, however, plant fungal pathogens can sporulate on previous years’ vegetation so no-till management regimes may increase disease pressures (Sharma-Poudyal et al., 2017). In addition to harboring pathogens on plant material, no-till management may allow diseases to persist by increasing soil moisture and slowing soil warming as demonstrated with plant-pathogenic oomycetes, such as *Pythium* and *Phytopthora* (Licht and Al-Kaisi, 2005; Broders et al., 2007). Under drought conditions, no-till corn and soybean crops have shown yield improvements, which has been attributed to increased soil moisture retention (Verhulst et al., 2011; Daigha et al., 2018). These factors and others may contribute to reports of increased grain yield for no-till managed soybean at several sites, including historically at the Kellogg Biological Station (KBS) Long Term Ecological Research (LTER) site (Pittelkow et al., 2015; Robertson et al., 2015).

In addition to reduced-tillage strategies, organic farming is another important alternative management strategy. In 2016, US organic soybeans were valued at more than $78 million US dollars (USDA, 2017). Acreage of organic field crops has increased since the 1990s, yet the share of total soybeans considered to be certified organic remained below 1% in 2015 (McBride and Greene, 2015). Although farmers must weigh the considerations mentioned above in determining management strategies, many soybean crops are managed with conventional tillage regimes. Tilling reduces plant material left in fields, which is a source of fungal disease propagules that then can be transferred to live plants; which has been demonstrated with *Rhizoctonia oryzae* (Schroeder and Paulitz, 2006).

It is also important to consider the effect of management systems on the plant and soil microbiome. Previous studies have investigated the effect of tillage regimes in conventional and organic wheat (Hartman et al., 2018) and corn (Wattenburger et al., 2019). These studies found that the management system influenced microbial community composition in roots and soils (Li et al., 2012; Hartman et al., 2018). In contrast, a whole plant microbiome study on root, stem, and leaf organs of wheat at the KBS-LTER found that the impact of management system was subtle (Gdanetz and Trail, 2017). Studies investigating the impact of management regime on the soybean microbiome have focused on specific bacterial taxa. One such study showed that conventional management reduces the diversity of *Rhizobium* populations associated with soybean (Hungria et al., 2006), while another study demonstrated that the relative abundance of Acidobacteria was reduced in soybean cultivated soils compared to forest soils (Navarrete et al., 2013).

The stage of plant growth at sampling is another important source of microbial community variation that has been observed in agricultural systems including biofuel crops and soybean (Sugiyama et al., 2014; Grady et al., 2019). For example, it was demonstrated that in the soybean rhizosphere, the relative abundance of *Bacillus, Rhizobium*, and *Bradyrhizobium* increased throughout the growing season (Li et al., 2012). In addition to composition shifts, a study on the wheat microbiome found that alpha diversity of prokaryotic communities increased throughout the growing season in both above and belowground plant tissues, but this trend was less clear for fungal communities (Gdanetz and Trail, 2017).

Here we characterize the fungal and prokaryotic communities, associated with individual soybean plants grown as part of a corn-soy-wheat rotation system under conventional, no-till, and organic management systems for nearly 30 years, to determine the impact of cropping management system on the soybean microbiome throughout a growing season. This study is part of a long term field experiment on the effect of agricultural management on plant and soil microbiomes in the corn-soy-wheat rotation at the KBS LTER, and follows previous research on the wheat-associated microbiome (Gdanetz and Trail, 2017). Although the present study is limited by representing a single site and season, results presented here will be available for future longitudinal microbiome studies from the same site under the consistent management provided by the KBS LTER. The organic management plots were planted with a non-genetically modified soybean variety to make it certified organic, while the no-till and conventional management plots were planted with a roundup ready genetically modified variety. Fungal and bacterial communities associated with soil, root, stem, and leaf compartments were characterized at three time points during the 2018 growing season. Management regime and plant developmental stage were hypothesized to impact the structure of the soybean microbiome. More specifically, we expected to see distinct differences between no-till and conventional/organic belowground microbial communities, due to microenvironment changes associated with tilling (Giller et al., 2015). In aboveground plant compartments, based on previous work done on wheat at the KBS LTER, we expected that variation in microbial communities would be primarily driven by growth stage (Gdanetz and Trail, 2017). To the best of our knowledge, this study represents the first characterization of the effect of agricultural management regime on the soybean microbiome in soil, roots, stems, and leaves across the growing season.

## Materials and Methods

### Sample Site and Management Systems

All samples were collected from the Michigan State University (MSU) W.K. KBS LTER crop rotation experiment in Hickory Corners, MI, United States. Soybean seeds were planted into one-hectare plots that have been managed under conventional, no-till, or organic management since 1989 (Robertson et al., 2015). Six replicate plots of each management system were distributed randomly at the LTER site in order to eliminate bias based on location.

The no-till and conventional management plots received fertilizer in the form of potash at a rate of 120 lbs/A (72 lbs/A K_2_O). In addition to fertilizer, plots within these two management systems received Valor herbicide treatments prior to emergence, at a rate of 3.5 oz/A (Valent Agriculture, United States). Additionally, the two management regimes received mid-season weed control with Roundup Powermax amended with ammonium sulfate at rates of 1 qt/A and 3.4 lbs/A, respectively (Bayer, Germany). Genetically modified soybean and corn have been grown at the LTER site since 2009 and 2011, respectively. The modified varieties provide glyphosate resistance as well as resistance to European corn borer and rootworm in corn (Robertson et al., 2015). During wheat rotation years, 30 pounds of nitrogen fertilizer/acre is applied to the conventional and no-till management systems in March as well as 43 pounds/A of nitrogen fertilizer and 25 pounds/A of sulfur fertilizer in May. Additionally, during wheat rotation years, conventional and no-till management plots receive herbicide applications in the form of Roundup PowerMax with ammonium sulfate in October and August (1 qt/A,3.4lbs/A) as well as Sharpen (2 oz/A), and corn methylated soybean oil (0.8 qt/A) in August (BASF, Germany; Van Dielst Supply Company, United States). During corn rotation years, nitrogen fertilizer is applied at planting at a rate of 29 lbs/A and in June at a rate of 122 lbs/A, and Lexar EZ herbicide is sprayed at a rate 3.0 qt/A alongside Roundup Powermax (22 oz/A) in June (Syngenta, United States). The certified organic management system received no chemical inputs or manure but was rotary hoed to control for weeds and has a red clover or annual rye cover crop in the winter season for all crops. The conventional and no-till management systems were planted with Pioneer P22T69R Roundup Ready soybean seed (Pioneer Hi Bred International, United States). The organically managed plots were planted with non-genetically modified Viking O.2188AT12N soybean seed (Albert Lea Seed, United States).

### Sampling and DNA Extraction Methods

In 2018, whole soybean plants were sampled at three time points corresponding to the following growth stages: early vegetative (V2 – two sets of unfolded trifoliate leaves), early reproductive (R2 – full flower inflorescence/reproductive stage), and late reproductive (R6 – full pod development) (Fehr et al., 1971). Within each management system (organic, no-till, conventional), three individual plants in each of four replicate plots were sampled at each of these growth stages (*n* = 108 plants). Throughout the growing season, samples from the organic management system were delayed 2 weeks due to later planting of the organic system. At each sampling point, independent samples of soil, roots, stems, and leaves were collected. Soil was sampled by removing whole plants from the soil and placing ∼2 g of soil from the root zone into a coin envelope which was then dried on silica beads upon return to the lab. Roots were sampled by cutting the entire root system at the soil line and placing the roots into a Whirl-Pak bag (Nasco, United States) containing a 0.1% Tween 20 mixture to remove soil before lyophilizing. The stem section between the first and second true leaves was collected in a 15 ml Falcon tube (Corning, United States) containing 5 mL of CSPL buffer from the Mag-Bind Plant DNA Plus Kit (Omega Bio-tek, United States). Leaves were sampled by hole punching three 6 mm leaf discs from three leaves into eppendorf tubes (Eppendorf, Germany) containing 500 μl of CSPL buffer. All samples were placed on ice and transported back to the Michigan State campus for storage at −80°C.

DNA was extracted from ∼50 mg of soil/sample using the PowerMag Soil DNA Isolation Kit (Qiagen, United States) on the KingFisher Flex system (Thermo Fisher Scientific, United States). DNA was extracted from ∼50 mg of each dried fine roots, stems, and leaves using the Mag-Bind Plant DNA Plus Kit (Omega Bio-tek, United States) on the KingFisher Flex system (Thermo Fisher Scientific, United States). All extractions included negative controls (extractions containing no sample).

### MiSeq Library Preparation and Sequencing

Illumina MiSeq amplicon libraries were constructed with the ITS1F – ITS4 primer set to target the internal transcribed spacer (ITS) region of Fungi and the 515F – 806R primer set to target the V4 region of the 16S rDNA of Prokaryotes (White et al., 1990; Gardes and Bruns, 1993; Caporaso et al., 2011). Libraries were prepared following a three step PCR protocol as described previously (Benucci et al., 2018, 2019; Chen K.H. et al., 2018). The PCR cycles used are shown in Supplementary Table S1. Unmodified primer pairs were used in the first step to enrich in target taxa. In the second step, primers incorporating frameshifts into the amplicons were used. In the third step, 10 nucleotide indexing barcodes and Illumina adapters were incorporated following approaches used by Chen K.H. et al. (2018) and Lundberg et al. (2013). PNA blocking clamps were incorporated into PCR reactions for steps one and two at a concentration of 0.75 μM to reduce the amplification of chloroplast and mitochondria sequences in plant-associated 16S libraries (PNA Bio Inc., United States). The PCR mixes used are shown in Supplementary Table S2. PCR products were run on an agarose gel to verify amplification. Next, PCR products were normalized to an equal concentration of 1–2 ng/μl using the SequalPrep Normalization Plate Kit (Thermo Fisher Scientific, United States). Following normalization, eluted samples were combined into one pool and concentrated with Amicon Ultra 0.5 mL 50K filters (EMD Millipore, Germany). Libraries were then cleaned with Agencourt AMPure XP magnetic beads to remove small fragments and primer dimers (Beckman Coulter, United States). Libraries were sequenced at the MSU Genomics Core with the Illumina Miseq V3 600 cycles kit. The produced sequences for the samples analyzed in this study are stored at the NCBI SRA archive under the following accession number: PRJNA603147. Sequences for samples that were not analyzed as part of this study, but were sequenced on the same Miseq runs and used for contaminant removal are available under the following accession numbers: PRJNA603199, PRJNA603207.

### Bioinformatics Analysis

First, sequences were analyzed for initial quality using FastQC^1^. Following quality analysis, reads were demultiplexed by barcode and assigned to samples using QIIME 1.9.1 (Caporaso et al., 2010). Due to lower quality of the reverse reads, only forward reads were analyzed further. Next, primers, adapters, and the conserved regions (SSU, 5.8S, LSU) of amplicons were stripped from forward sequences using Cutadapt v2.6 and USEARCH v10 (Martin, 2011; Edgar and Flyvbjerg, 2015; Edgar, 2016b). Afterward, library statistics were analyzed using USEARCH for length and quality distributions and reads below 205 bp and above a maximum error of 1% were discarded. Additionally, sequences were de-replicated and singletons were removed prior to clustering Operational Taxonomic Units (OTUs) at a 97% threshold using the UPARSE algorithm of USEARCH (Edgar, 2013, 2016b; Edgar and Flyvbjerg, 2015). Following OTU clustering, taxonomy was assigned to fungal OTUs using the UNITE database V10.10.2017 (Kõljalg et al., 2005) and 16S OTUs using the Silva 16S V123 database (Quast et al., 2013) with the SINTAX tool (Edgar, 2016a).

### Statistical Analyses

OTU tables, taxonomy tables, mapping files, and OTU sequences were loaded into the R (Version 3.5.2) statistical environment (R Core Team, 2018) and used to create a phyloseq object for further analysis in the *phyloseq* package (McMurdie and Holmes, 2013). Before analyzing sequence data, OTUs determined to be contaminants in negative controls were removed with the *decontam* package (Davis et al., 2018). Samples which produced less than 1000 reads, as well as five soil samples that did not dry properly and were overtaken by mold, were discarded. Alpha diversity (within sample diversity) was estimated for each sample before data was normalized and filtered following recommendations in McMurdie and Holmes (2014). Alpha diversity was estimated using richness (Simpson, 1949) and Shannon diversity (Hill, 1973) within the *BiodiversityR* and *vegan* packages (Kindt and Coe, 2005; Oksanen et al., 2019). OTU richness and Shannon diversity were visualized for each plant compartment with boxplots in *ggplot2* (Wickham, 2016). Differences in alpha diversity means due to management system, growth stage, and plant compartment were tested for statistical significance using Kruskal Wallis tests in the stats package (R Core Team, 2018). In the case of a significant result (*P* < 0.05), Pairwise Wilcox tests with a false discovery rate (FDR) *P*-value correction were utilized to determine significance groups by growth stage and management regime (R Core Team, 2018). Significance groups for growth stage and management system are denoted on alpha diversity boxplots by letters above boxes where significant differences (*P* < 0.05) were present between means of the same growth stage or the same management system. Following alpha diversity analyses, OTUs with less than five reads in a single sample were placed to zero to account for tag switching and OTUs with less than 10 reads across all samples were removed to account for PCR errors (Lindahl et al., 2013; Oliver et al., 2015). Rarefaction curves were created to assess the sampling of prokaryotic and fungal communities using the “rarecurve” function in the *vegan* package (Oksanen et al., 2019). Barplots for fungal communities were created in *ggplot2* to show genera having >4% relative abundance (Wickham, 2016); prokaryotic barplots were created to show genera (classes for soil) having >2% relative abundance. Indicator species analysis was performed with the *indicspecies* package to identify taxa which were significantly associated with either one single management system and not the other two or significantly associated with two of three management systems (De Cáceres and Legendre, 2009). Following identification of indicator OTUs, *p*-values were FDR adjusted, and only taxa with adjusted *p* < 0.05 were considered to be indicators. The top 30 most abundant identified indicator taxa were used to create heatmaps displaying the relative abundance distributions by management regime and growth stage of identified taxa in the *ComplexHeatmap* package in R (Gu et al., 2016).

Next, data were normalized by cumulative sum scaling in the *metagenomeseq* package (Paulson et al., 2013). Following normalization, beta diversity was analyzed in the *phyloseq* and *vegan* packages by creating Principal Coordinates Analysis (PCoA) plots with the “ordinate” and “plot_ordination” functions. Community patterns identified in PCoA plots were tested for statistical significance using PERMANOVA as implemented by the “adonis” function in vegan. Homogeneity of variance between modeled groups was analyzed with the “betadisper” function in *vegan*. To further assess microbial community differences between management systems, random forest models were created to test the accuracy of assigning above and belowground samples to their management system origin using the “randomforest” function in the *randomForest* package in R (Liaw and Wiener, 2002). Random forest models were optimized by testing different mtry values (number of OTUs randomly sampled from the community to build models). Mtry values of ±10 of the standard value (square root of the number of OTUs in the community) were tested. If the out of bag error did not improve any tested mtry values, the standard value was used. Figures were created from the results of random forest models, displaying the following: the out of bag error plotted against the number of trees, MDS plots created from random forest sample proximities converted to Bray-Curtis distances, and the top 30 OTUs important in assigning samples to their management system. Importance of each individual OTU for distinguishing between management systems was assessed by calculating the mean decrease in model accuracy when that OTU is removed from the community. Significance of random forest models was tested with 999 permutations (random forest models were repeated 999 times) using the “rf.significance” function in the *rfUtilities* package in R (Murphy et al., 2010).

Bipartite co-occurrence networks containing both bacteria and Fungi were created and analyzed using the *SpiecEasi* and *Igraph* packages in R (Csardi and Nepusz, 2006; Kurtz et al., 2015). Networks were constructed with OTUs that were present in 80% of samples or more. Network stability and sparsity were assessed using *SpiecEasi*. Hub taxa were identified as those above the 90th percentile (1.3 standard deviations from the mean) of network OTUs for the measures of degree and betweenness centrality as outlined in Agler et al. (2016). Additionally, taxa were only considered to be hubs if they were above the 90th percentile of hub scores (eigenvector centrality) for either Fungi or Bacteria in that specific network. The betweenness centrality measure was log transformed before determining hubs to account for a non-normal distribution. Following network creation in *Spieceasi* and hub identification, networks were visualized with the attribute circular layout in the Cytoscape program (Shannon et al., 2003). Random networks with the same number of nodes as experimental networks were generated with the Barbasi-Albert model of the “sample_pa” function in the *igraph* package of R. The degree distributions of 100 random networks were compared to those of experimental networks with a two sample Kolmogorov-Smirnov using the “ks.test” function in the *stats* package of R. All R code and files for producing figures and tables including metadata and OTU tables, as well as example code for building networks and random forest models is available at: https://github.com/longleyr/Management-of-Soybean-Code-and-Files.

## Results

### Next Generation Sequencing Results

The final soil fungal library contained 95 samples and 2,562,324 reads for an average depth of 26,972 reads per sample after filtering, removal of contaminants, and removal of samples with less than 1000 reads. Applying the same quality filtering by plant compartment, the root fungal library was composed of 100 samples containing 2,706,574 reads with an average depth of 27,618 reads per sample, the stem fungal library contained 618,697 reads in 93 samples with an average depth of 7,031 reads per sample, and the library for the leaves had 4,572,077 reads in 107 samples for an average read depth of 43,133 reads per sample. Applying these quality filtering criteria to prokaryotic communities the 16S marker produced 6,040,145 reads with an average depth of 59,217 reads in 102 soil samples, 6,378,213 16S reads with an average depth of 60,172 reads from 106 root samples, 1,435,193 reads with an average depth of 14,497 reads per sample from 99 stem samples, and 1,313,368 reads with an average depth 13,402 reads per sample in 99 leaf samples. Rarefaction curves showing the number of OTUs generated against sequencing depth for each sample are shown in Supplementary Figure S1.

### Fungal Community Composition

**FIGURE 1:**
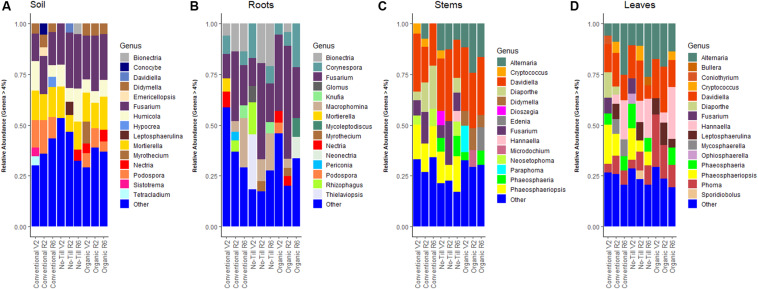
Stacked bar plots showing fungal genera in each management system at each growth stage (V2 – two sets of unfolded trifoliate leaves, R2 – full flower reproductive stage, and R6 – full pod development) with relative abundance ≥4%, **(A)** present in soil samples throughout the soybean growing season, **(B)** present in soybean root samples, **(C)** present in soybean stem samples, and **(D)** present in soybean leaf samples.

In the soil, Ascomycota were dominant, independent of management system, and accounted for between 75.0 and 81.0% of total reads. In comparison, Mucoromycota and Basidiomycota abundances ranged between 7.0 and 12.9% in the three management systems (conventional, no-till, and organic). Of note, *Fusarium* was the most abundant fungal genus in the soil across all management systems with a relative abundance range of 15.9–23.7% (Figure 1A). All management regimes also contained a high abundance of *Mortierella* in soils with a range of 12.5–14.3%. Ascomycota dominated the fungal community of the roots under all three management systems, accounting for between 82.2 and 85.0% of reads, Glomeromycotina (8.3–13.5%) was the next most abundant lineage. Basidiomycota was present at relative abundances of between 3.7 and 8.1% in the three management systems. As found in the soil, the most abundant genus under all three management systems was *Fusarium*, which represented between 22.1 and 37.7% of all reads (Figure 1B). *Fusarium* was followed in relative abundance by *Macrophomina* in the conventional management system (13.2%), *Bionectria* in the no-till management system (13.8%) and *Corynespora* in the organic management system (11.7%).

In stems, Ascomycota and Basidiomycota accounted for nearly 100% of reads in all management regimes with Ascomycota accounting for about 90.0% of the reads. *Davidiella* was the most abundant genus in the stems, with over 20.0% of the reads in all three management systems followed by *Diaporthe* in conventionally managed plots and *Fusarium* and *Alternaria* in no-till and organic management systems (Figure 1C). As was found in the stems, Ascomycota and Basidiomycota accounted for nearly 100% of the reads in the leaves of each management system; with ascomycetes accounting for ∼75.0% of the reads. *Alternaria* was abundant in aboveground tissues of all management regimes and was the most abundant genus in the conventional and no-till management systems, with relative abundances of 14.9 and 15.5%, respectively. *Davidiella* was omnipresent in aboveground tissues, peaking in relative abundance at 20.0% in the organic management system. This was also true of *Phoma*, which had higher relative abundance in the organic management regime (Figure 1D).

### Prokaryotic Community Composition

**FIGURE 2:**
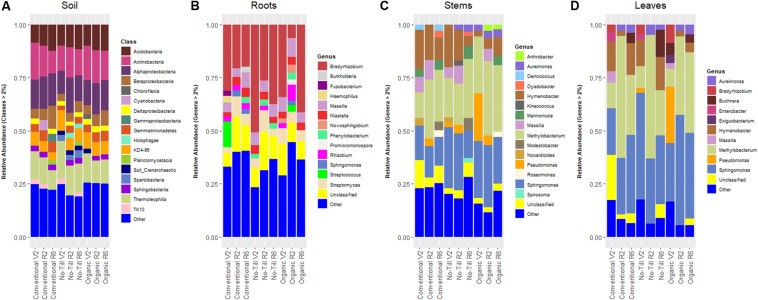
Stacked bar plots showing prokaryotic classes or genera in each management system at each growth stage (V2 – two sets of unfolded trifoliate leaves, R2 – full flower reproductive stage, and R6 – full pod development) with relative abundance ≥2%, **(A)** present in soil samples in soybean fields throughout the growing season, **(B)** present in soybean root samples, **(C)** present in soybean stem samples, and **(D)** present in soybean leaf samples.

The prokaryotic community of the soil was relatively consistent across management systems in terms of dominant Phyla. The most abundant phylum in every management system was Actinobacteria, consistently represented by ∼30% relative abundance. The next most dominant phylum in each management system was Proteobacteria having relative abundances between 20.0 and 24.0%. In the soil, the most abundant classes were consistent between managements, but differed in their relative abundances (Figure 2A). The most abundant genus in every management system was an unclassified member of the Chloroflexi phylum with a range of relative abundances between 5.5 and 7.4%. *Sphingomonas* was the second most abundant genus (4.8%) in conventional managed soils. In contrast, an unclassified Gaiellales genus (6.7%) was the second most abundant in the no-till, while an unidentified genus of acidobacteria was the second most abundant in the organically managed soils. Soybean roots were dominated by the same bacteria phyla as the soils, but Proteobacteria were more abundant in roots (57.3–71.7%) compared to 20.0–24.1% in soil. Actinobacteria were the second most abundant bacteria in soybean roots (17.1–21.1%) across management systems. The most abundant genus was *Bradyrhizobium* with relative abundances of 22.9, 40.2, and 33.0% in the conventional, no-till, and organic management regimes, respectively. Following *Bradyrhizobium*, *Streptomyces* was the next highest in relative abundance ranging between 6.4 and 7.1% (Figure 2B).

The stem prokaryotic community was also dominated by Proteobacteria, with relative abundances ranging from 60.0 to 77.0%. Actinobacteria were the second most abundant bacteria in no-till (20.8%) and organic (12.7%) management systems. In terms of genera, the stems of soybean in all three management systems were dominated by *Methylobacterium* (24.3–32.0%) and *Sphingomonas* (14.9–25.2%) (Figure 2C). The prokaryotic community of soybean leaves was quite like that of the stems. Proteobacteria dominated the community ranging from 78.2% in the conventional management system to 92.6% in the organic management system. The dominant genera in leaves were similar to the stems except that *Sphingomonas* had higher relative abundance in the leaves, ranging from 31.5 to 44.7%. The relative abundance of *Methylobacterium* in the leaves was between 28.1 and 36.1% (Figure 2D).

### Alpha Diversity of Fungal Communities

**FIGURE 3:**
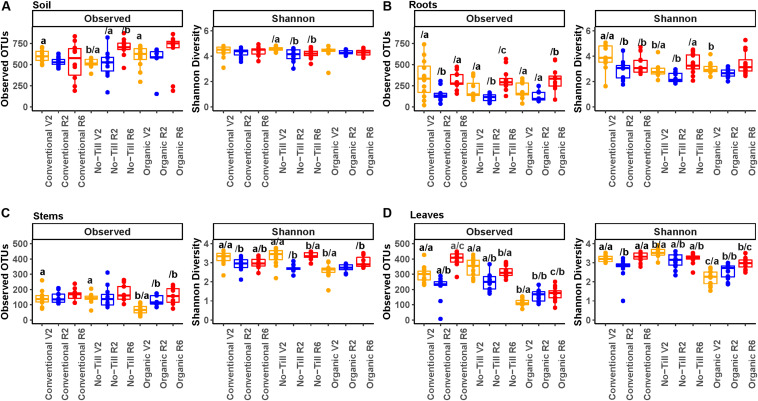
Alpha diversity boxplots showing OTU richness and Shannon diversity metrics for fungal communities, **(A)** present in soil samples, **(B)** present in soybean root samples, **(C)** present in soybean stem samples, and **(D)** present in soybean leaf samples. Colors represent the plant growth stage during sampling (V2 – two sets of unfolded trifoliate leaves, R2 – full flower reproductive stage, and R6 – full pod development). Significance groups are represented by letters above the boxes. The letter before the forward slash (/) represents significance groups within a single growth stage by management system. Letters following the forward slash (/) represent significance groups within a single management system by growth stage. Significance groups were calculated using Kruskal Wallis tests followed by Pairwise Wilcox tests with a FDR *P*-value correction.

Differences in fungal alpha diversity due to management system, plant growth stage, or sample origin were assessed. Fungal alpha diversity was highest in the soil and lowest in the stems with roots and leaves falling between the two (Figure 3). Soil had significantly higher species richness compared to roots, leaves, and stems (579 taxa per sample, 237 taxa per sample, 252 taxa per sample, and 140 taxa per sample, respectively). Richness differences between roots and leaves were not significant, but they both had significantly greater richness than stems. The soil also had significantly higher Shannon diversity than roots, stems, and leaves but differences between plant compartments were non-significant. In the soil, the only significant difference in richness between management systems was between the conventional/organic and no-till management systems at the V2 growth stage (Figure 3A). Significant differences were detected by growth stage under the no-till management system, with significantly higher fungal richness in the final growth stage but a decrease in Shannon diversity. In the root microbiome, there were significant differences in Shannon diversity at the early vegetative (V2) growth stage with the organic management regime having significantly lower Shannon diversity mean values (Figure 3B). All management systems showed a decrease in fungal richness and Shannon diversity at the early reproductive (R2) stage, which increased again at the late reproductive (R6) stage.

In soybean stems, the conventional and no-till management systems consistently had higher richness than the organic management system, but the difference was only significant at the early vegetative (V2) growth stage (Figure 3C). This trend was not consistently reflected in Shannon diversity. All three management systems showed increasing richness throughout the season in the stems, but the trend was only significant for the organic management regime. Alpha diversity trends in the leaves of soybean in each management system were similar to those of their stems, with significantly greater richness in the conventional and no-till management systems throughout the experiment (Figure 3D). Fungal richness increased throughout the experiment in organic treatments, but in the other management systems richness and Shannon diversity decreased at the early reproductive (R2) growth stage.

### Alpha Diversity of Prokaryotic Communities

**FIGURE 4:**
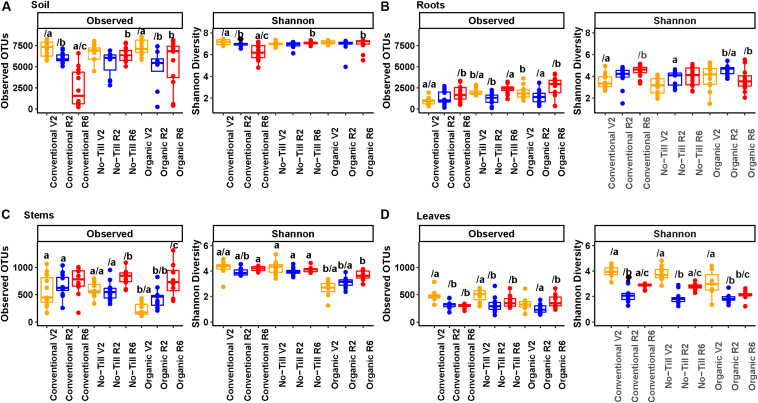
Alpha diversity boxplots showing OTU richness and Shannon diversity metrics for prokaryotic communities, **(A)** present in soil samples, **(B)** present in soybean root samples, **(C)** present in soybean stem samples, and **(D)** present in soybean leaf samples. Colors represent the plant growth stage during sampling (V2 – two sets of unfolded trifoliate leaves, R2 – full flower reproductive stage, and R6 – full pod development). Significance groups are represented by letters above the boxes. The letter before the forward slash (/) represents significance groups within a single growth stage by management system. Letters following the forward slash (/) represent significance groups within a single management system by growth stage. Significance groups were calculated using Kruskal Wallis tests followed by Pairwise Wilcox tests with a FDR *P*-value correction.

In prokaryotic communities, OTU richness was highest in the soil and decreased moving from that of the roots toward distal aerial compartments (Figure 4). Soil alpha diversity was significantly greater than the roots, stems, and leaves (5780 OTUs per sample, 1761 OTUs per sample, 597 OTUs per sample, and 358 per sample, respectively). Additionally, the roots had significantly greater alpha diversity compared to stems and leaves, but differences between stems and leaves were not significant. This pattern of statistical significance also held true for Shannon diversity, with a range from 2.7 in the leaves to 6.9 in the soil. In terms of Shannon diversity, differences between roots and stems were not significant (3.88 and 3.80, respectively). In the soil, at any single growth stage, there were no significant differences between management systems except at the final stage where the conventional management system had significantly lower richness and Shannon diversity compared to the other management systems (Figure 4A). Conventional and organic management regimes showed significantly lower richness in the reproductive stages compared to the vegetative (V2) stage. In the roots, the richness was significantly lower in the conventional management system at the first sampling point, but differences were not significant at later stages (Figure 4B). The no-till and organic management systems showed significant decreases in richness at the R2 growth stage, but this pattern was not reflected in Shannon diversity.

In the stems, the no-till management system had significantly lower richness in the first growth stage compared to the final stage, and Shannon diversity was significantly lower in the organic management system throughout the season compared to other management systems (Figure 4C). Richness increased between the first and last sampling point for all three management systems, but this change was only significant for no-till and organic management regimes. In the leaves, the organic management system had lower richness and Shannon diversity at the early vegetative (V2) growth stage, but this difference was not significant. All three management systems had a significant decrease in richness and Shannon diversity in the leaves at the early reproductive (R2) growth stage (Figure 4D).

### Beta Diversity of Fungal Communities

**FIGURE 5:**
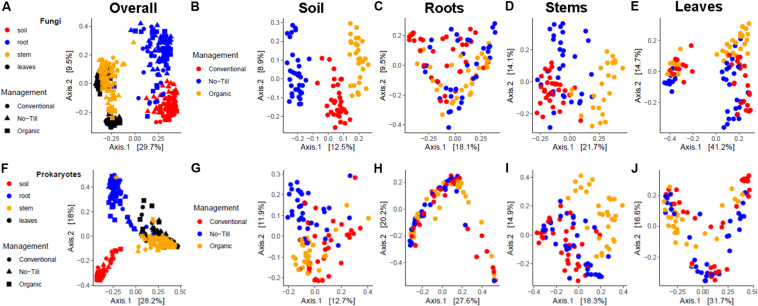
Principal coordinates analysis plots, based on Bray-Curtis dissimilarity, of fungal communities, **(A)** associated with soybean soil, root, stem, and leaf samples, **(B)** associated with soil samples, **(C)** associated with root samples, **(D)** associated with stem samples, **(E)** associated with leaf samples and prokaryotic, **(F)** associated with soil, root, stem, and leaf samples, **(G)** associated with soil samples, **(H)** associated with root samples, **(I)** associated with stem samples, and **(J)** associated with leaf samples. The shape represents the management system, while color represents sample origin in **(A,F)**. In all others the color represents the management system.

When considering all sampling sources together, the soybean-associated fungal communities were most separated by sample source (Figure 5A). When considering PCoA ordinations by individual sample origin, distinct clusters by management system are evident in the soil (Figure 5B). In the stems and the leaves there is some separation by the management system along both axes, but the management systems are not distinct (Figures 5D,E). There is no clear pattern among root samples by PCoA (Figure 5C). When samples are colored by growth stage, there are distinct clusters by growth stage along the X axis in the leaves. This axis accounts for 41% of the variation and primarily separates the V2 growth stage on the left from the R2 and R6 growth stages (Supplementary Figure S2E). There was some clustering by growth stage in the stems, but clusters were not as distinct compared to the leaves (Supplementary Figure S2D). There was no clear pattern of fungal communities by growth stage in the soil or roots (Supplementary Figures S2B,C).

The PERMANOVA analysis of fungal communities showed that regardless of sample origin there was a significant (*P* < 0.05) effect of both management system and growth stage (Supplementary Table S3A). However, since there was also a significant (*p* < 0.05) effect of the interaction between management regime and growth stage, datasets were split by growth stage and management system to analyze the effects separately (Table 1 and Supplementary Table S3). When split by growth stage, the effect of management system, was significant across all growth stages and all plant organs (Table 1A). This effect accounted for between 13 and 52% of variation. However at several growth stages in several sample origins, there was a significant effect of dispersion, confounding PERMANOVA results (Soil R2 – *P*-value: 0.0096, Soil R6 – *P*-value: 0.023, Roots R2 – *P*-value: 0.0027, Leaves V2 – *P*-value: 0.0027). Although there is a significant effect of dispersion for these groups, there is clustering by management system in the PCoA ordination space for the soil and the leaves, but clustering is less clear for R2 roots (Supplementary Figures S3A,C,G). When split into individual management systems, the effect of growth stage is also significant throughout all management regimes and all plant compartments (Table 1B). The no-till roots and the conventional leaves have significant differences in group dispersion (*P*-values 0.042 and 0.037, respectively), but there do appear to be distinct clusters by growth stage in the ordination space for these groups (Supplementary Figures S3D,H).

**TABLE 1:** Permutational multivariate analysis of variance *(adonis)* and multivariate homogeneity of group dispersion analysis *(betadisper)* results for fungal communities associated with soybean soil, root, stem, and leaf samples showing, (A) the effect of agricultural management on individual growth stages (V2 – two sets of unfolded trifoliate leaves, R2 – full flower reproductive stage, and R6 – full pod development), and (B) the effect of growth stage on individual agricultural management systems.

**A – Growth stage**		**PERMANOVA**			**DISPERSION**	

		***F*-value**	**R^2^**	***P*-value**	***F*-value**	***P*-value**
Soil	V2	5.23	0.24	**1.00E-04**	3.08	0.052
	R2	4.001	0.24	**1.00E-04**	5.63	**0.0096**
	R6	4.59	0.25	**1.00E-04**	3.63	**0.0396**
Roots	V2	3.6	0.19	**1.00E-04**	0.669	0.519
	R2	2.08	0.14	**5.90E-03**	4.39	**0.023**
	R6	2.85	0.15	**1.00E-04**	0.145	0.866
Stems	V2	4.89	0.26	**1.00E-04**	0.709	0.501
	R2	4.2	0.24	**1.00E-04**	3.15	0.059
	R6	9.98	0.44	**1.00E-04**	1.57	0.227
Leaves	V2	17.6	0.52	**1.00E-04**	7.15	**0.0027**
	R2	5.86	0.27	**1.00E-04**	1.34	0.276
	R6	7.74	0.32	**1.00E-04**	3.27	0.051

**B – Management**		**PERMANOVA**			**DISPERSION**	

		***F*-value**	**R^2^**	***P*-value**	***F*-value**	***P*-value**

Soil	Conventional	1.62	0.09	**8.00E-04**	0.728	0.491
	No-till	2.72	0.14	**1.00E-04**	0.903	0.416
	Organic	1.65	0.11	**0.0065**	0.0401	0.961
Roots	Conventional	2.83	0.16	**1.00E-04**	0.786	0.465
	No-till	4.18	0.20	**1.00E-04**	3.49	**0.042**
	Organic	2.29	0.14	**1.60E-03**	2.86	0.074
Stems	Conventional	3.67	0.19	**1.00E-04**	1.39	0.264
	No-till	5.21	0.26	**1.00E-04**	1.19	0.318
	Organic	2.73	0.21	**1.00E-04**	1.77	0.196
Leaves	Conventional	27.6	0.64	**1.00E-04**	3.66	**0.037**
	No-till	29.1	0.63	**1.00E-04**	1.24	0.302
	Organic	19.9	0.54	**1.00E-04**	3.23	0.052

*Significant *P*-values (*P* < 0.05) are shown in bold.*

Beta diversity of no-till and conventional management systems were analyzed together without the organic management regime due to the difference of host genotype. There was a significant effect of management system on beta diversity across all plant compartments and all growth stages with the effect ranging from 9 to 29% (Supplementary Table S4 and Supplementary Figure S5). When split into no-till and conventional management systems, the effect of growth stage was also significant across management systems and sample origins. In the no-till roots, a significant effect of group dispersion (*P* = 0.016) was found, with separation of growth stages obvious in ordinational space (Supplementary Figure S5D).

### Beta Diversity of Prokaryotic Communities

When all samples are considered together, prokaryotic communities are clustered by sample origin, although there was not a clear distinction between stems and leaves (Figure 5F). When separated by sample origin, there were not clear clusters by management regime in any sample origin, but in the soil the no-till management system did appear slightly separated from the conventional and organic, primarily appearing in the upper left of the ordinational space (Figure 5G). In the stems, the organic management system was the most distinct, primarily appearing in the upper right of the PCoA (Figure 5I). When samples are colored by growth stage, there are clear clusters for each growth stage in the stem and leaf PCoAs with separation along the X and Y axes (Supplementary Figures S2I,J). Similarly to fungal communities, soil and root prokaryotic communities did not show distinct clusters by plant growth stage.

Growth stage and management system had a significant effect (*P* < 0.05) on prokaryotic communities at all sample origins, and the effect of plant growth stage increased moving upwards from the soil to aboveground and distal compartments of the plant (Supplementary Table S3B). Since there were significant interactions between growth stage and management system as well as significant differences in group dispersion, datasets were split by management regime and growth stage and analyzed separately (Table 2 and Supplementary Figure S6). When split by growth stage, the effect of management system is significant across all sample origins and all growth stages. This effect accounts for between 11.3% (R2 roots) and 30.1% (R2 stems) of the variation. In several groups, there was a significant effect of group dispersion, making PERMANOVA results difficult to interpret. In the soil, at all three growth stages there was a significant (*P* = 0.00037, 0.0417, 0.00271) effect of dispersion, but in the ordinational space, there does seem to be separation by management system (Supplementary Figure S6A). In the V2 leaves, where there was also a significant effect of dispersion (*P* = 0.045), distinct clusters by management system are visible in the PCoA (Supplementary Figure S6G). When split by management system, there was a significant effect of growth stage in all management systems and all sample origins. This effect accounted for the most variation in the leaves where it accounted for between 42 and 53% of variation (Table 2B). However, there was a significant effect of group dispersion (*P* = 0.0013, 0.0041, 0.00073) in the leaves in all management systems, but samples do cluster by growth stage in the ordinational space (Supplementary Figure S6G). In conventional soil and organic stems, there is also a significant effect of group dispersion (*P* = 2.5E-8, 7.2 E-5, respectively), but separation by growth stage is less clear in the ordinational space (Supplementary Figures S6B,F).

**TABLE 2:** Permutational multivariate analysis of variance *adonis* and multivariate homogeneity of groups dispersions analysis *(betadisper)* results for prokaryotic communities associated with soybean soil, root, stem, and leal samples showing (A) the effect of agricultural management on individual growth stages (V2 – two sets of unfolded tri foliate leaves, R2 – full flower reproductive stage, and R6 – full pod development), and (B) the effect of growth stage on individual agricultural management systems.

**A – Growth stage**		**PERMANOVA**			**DISPERSION**	

		***F*-value**	**R^2^**	***P*-value**	***F*-value**	***P*-value**
Soil	V2	5.93	0.26	**1.00E-04**	10.1	**3.70E-04**
	R2	3.47	0.21	**1.00E-04**	3.6	**0.0417**
	R6	3.1	0.16	**1.00E-04**	7.11	**2.71E-03**
Roots	V2	3.56	0.18	**2.00E-04**	2.07	0.143
	R2	2.01	0.11	**0.0123**	0.032	0.969
	R6	3.32	0.18	**1.90E-03**	0.739	0.486
Stems	V2	6.17	0.29	**1.00E-04**	3.44	**0.045**
	R2	6.9	0.30	**1.00E-04**	0.247	0.783
	R6	4.49	0.25	**1.00E-04**	1.11	0.344
Leaves	V2	3.94	0.23	**1.00E-04**	3.49	**0.045**
	R2	3.29	0.18	**1.00E-04**	0.119	0.887
	R6	6.25	0.28	**1.00E-04**	0.289	0.751

**B – Management**		**PERMANOVA**			**DISPERSION**	

		***F*-value**	**R^2^**	***P*-value**	***F*-value**	***P*-value**

Soil	Conventional	3.1	0.16	**1.00E-04**	31.7	**2.50E-08**
	No-Till	2.21	0.12	**1.10E-03**	1.01	0.376
	Organic	2.55	0.18	**3.00E-04**	0.49	0.619
Roots	Conventional	2.34	0.13	**7.20E-03**	0.175	0.841
	No-Till	4.09	0.21	**1.00E-04**	0.898	0.418
	Organic	3.76	0.19	**1.00E-04**	0.796	0.459
Stems	Conventional	5.2	0.26	**1.00E-04**	1.04	0.367
	No-Till	9.1	0.36	**1.00E-04**	1.07	0.356
	Organic	4.31	0.24	**2.00E-04**	13.9	**7.20E-05**
Leaves	Conventional	13.9	0.48	**1.00E-04**	8.26	**1.30E-03**
	No-Till	18.6	0.54	**1.00E-04**	6.55	**4.12E-03**
	Organic	9.85	0.42	**1.00E-04**	9.56	**7.30E-04**

*Significant *P*-values (*P* < 0.05) are shown in bold.*

As with fungal communities, the no-till and conventional systems were analyzed without the organic system due to the genotypic difference. When split into individual growth stages, the effect of management system is significant (*p* < 0.05) in all growth stages and all sample origins except R2 roots and R2 leaves. This effect is the largest in the V2 soil and the V2 leaves (22.8 and 20.3% respectively). In groups where the management system effect is significant, distinct clusters are apparent in the ordinational spaces (Supplementary Figure S7), although clusters are less distinct than those of Fungi. In the R6 soil, there was a significant effect of group dispersion (*P* = 4.58E-4), but the PCoA reveals separation between no-till and conventional management systems. When split into no-till and conventional management regimes, the effect of growth stage was significant throughout the management systems and sample origins. In the leaves, there were significant differences (*P* = 0.0012, 0.0097) in group dispersion for both conventional and no-till management systems, but there are distinct clusters by growth stage in both management regimes (Supplementary Figure S7H).

### Indicator Species Analysis and Random Forest Modeling of Fungal Communities

**FIGURE 6:**
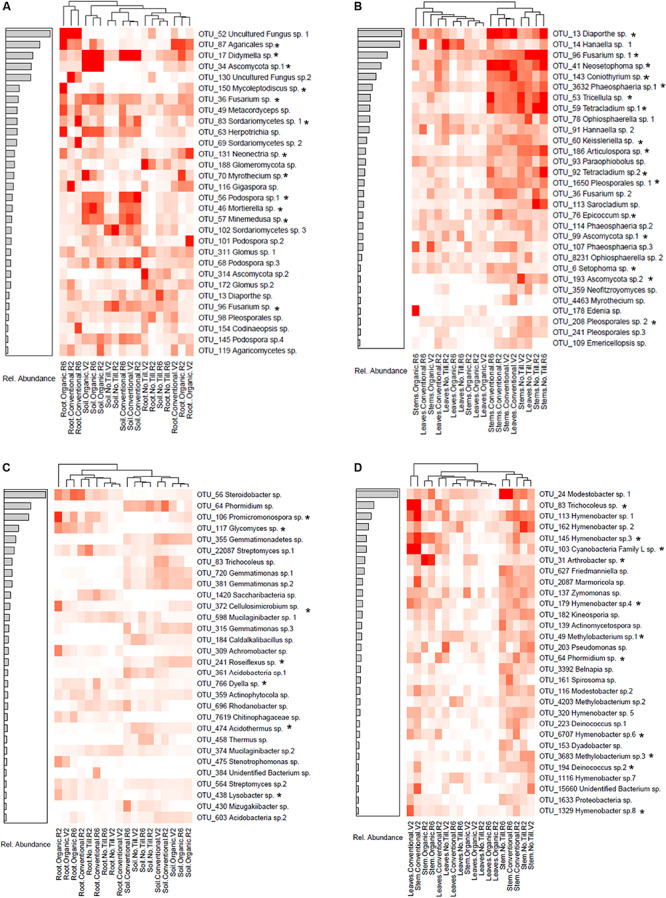
Heatmaps of the relative abundances of the top 30 most abundant indicator taxa of fungal **(A)** belowground taxa, **(B)** aboveground taxa and prokaryotic **(C)** belowground taxa, and **(D)** aboveground taxa associated with conventional, no-till, organic, conventional and no-till, conventional and organic, or no-till and organic management systems. Samples are clustered by the displayed dendrogram using Bray-Curtis distances. The associated barplots show the relative abundance among indicator species of the taxa. Taxa that were also among the top 30 most important for distinguishing between managements in Random Forest models of above and belowground samples are indicated with an asterisk (*).

Many fungal OTUs were strongly associated with specific management systems. Heatmaps of the top 30 most relatively abundant indicator OTUs in above and belowground samples are shown in Figure 6. In belowground fungal communities, many of the indicator taxa were OTUs which were indicators for conventional and organic soils but were lacking in no-till soils. These indicators were from several genera including *Didymella* OTU 17, *Mortierella* OTU 46, *Podospora* OTU 56, and *Minemedusa* OTU 57 (Figure 6A). All these taxa were also identified as being in the top 30 most important taxa for distinguishing between management systems in random forest analysis (Supplementary Figure S4A). Indicators to no-till soils included a Sordariomycetes OTU and *Fusarium* OTU 96 which was also identified by random forest analysis. In the roots, a Glomeromycotina OTU 188 was highly associated to the no-till management system. An unidentified Agaricales OTU 87 was an indicator for the conventional and organic management regimes, and *Mycoleptodiscus* OTU 150 was an indicator for organic root communities (Figure 6A). Both taxa were also identified in random forest models as being important in distinguishing between management systems.

In aboveground fungal communities, many of the indicator species for no-till and conventional stems and early vegetative (V2) leaves clustered together. These OTUs included *Diaporthe* OTU 13 which was abundant in conventional stems and leaves, as well as *Fusarium* OTU 96 which was abundant in no-till tissues. Both indicator taxa were also identified as being important in aboveground random forest models. At later growth stages in the leaves, a *Hanaella* sp. was an indicator in the conventional and no-till leaves. The main indicator for the organic management system was an *Edenia* sp. which was most highly abundant in the stems at the late reproductive (R6) growth stage (Figure 6B).

Trends identified through indicator species analysis were further assessed with random forest analysis. Above and belowground fungal communities were assessed, and it was demonstrated that for belowground fungal communities there was an out of bag error for assigning management system to belowground samples of 7.7% (Supplementary Figure S4A). Conventional samples were assigned incorrectly 10.6% of the time, no-till samples were assigned incorrectly 4.3% of the time, and organic samples were assigned incorrectly 10% of the time. For aboveground samples, the out of bag error was 3.1% (Supplementary Figure S4B). The error rate in the organic management system was 0.0%, while the rate for conventional samples was 1.4% and the rate for no-till samples was 8.8%. Conversion of sample proximities to Bray-Curtis distance allowed for the visualization of clusters of samples by each management regime for above and belowground samples (Supplementary Figures S4A,B). Random forest models identified several *Phoma* and *Paraphoma* taxa which were important in distinguishing management systems but were not identified by indicator species analysis (Supplementary Figure S4A).

### Indicator Species Analysis and Random Forest Modeling of Prokaryotic Communities

Belowground prokaryotic indicator OTUs in root and soil compartments form into groups when clustered by Bray-Curtis distances. In the organic management system, *Steroidobacter* OTU 56 and *Promicromonospora* OTU 106 were indicator Bacteria in the soybean root compartment, whereas a *Streptomyces* OTU was an indicator in roots from conventional and no-till management systems. *Promicromonsopora* OTU 106 was also identified as being among the top 30 most important OTUs in random forest modeling (Supplementary Figure S4C). In the soil, a *Phormidium* OTU was an indicator for conventional and no-till management systems, while *Cellulosmicrobium* OTU 372 was an indicator to the organic soil and roots and was also identified by random forest modeling (Figure 6C and Supplementary Figure S4C).

In aboveground tissues, indicator OTUs clustered based on plant compartments, management regime and growth stage. For example, at the early vegetative (V2) growth stage, stems and leaves from conventional managed soybean shared several indicator OTUs, including Cyanobacteria belonging to *Tricholeus* (OTU 83) and an unidentified Cyanobacteria Family L species (OTU 103). Both taxa were also identified as being important for assigning samples to management systems by random forest analysis. Many of the bacterial indicator taxa were *Hymenobacter* species, the majority of which were associated with no-till and conventional management regimes in both leaves and stems (Figure 6D). Many of the *Hymenobacter* taxa were also identified as being important in random forest modeling (Supplementary Figure S4D). A stem associated *Arthrobacter* sp. was an indicator of the organic management system and was identified in random forest modeling.

Random forest modeling performed on belowground prokaryotic communities revealed that samples were assigned to the correct management system 89.9% of the time (Supplementary Figure S4C). Samples of the conventional management regime were assigned incorrectly 10.1% of the time, no-till samples 9.9% of the time, and organic samples 10.3% of the time. When proximities between samples were converted to Bray Curtis distance, clustering by management system is visible, but less clear compared to belowground fungal communities. The aboveground prokaryotic random forest model had an out of bag error rate of 10.7% (Supplementary Figure S4D). The conventional management system samples were assigned incorrectly 12.1% of the time, no-till samples were assigned incorrectly 4.2% of the time, while organic samples were assigned incorrectly 0% of the time. In the MDS space, there was separation by management system, but the clusters were less clear than aboveground Fungi (Supplementary Figure S4D).

### Core Network Analysis and Hub Species Detection

Microbial networks constructed for above and belowground compartments across each management system differed in their network statistics (Table 3). Microbial networks in the no-till management system had the greatest numbers of nodes and edges for both above and belowground networks. Belowground, the network for the organic management system had the next highest number of edges and nodes, but aboveground the organic network was the sparsest in terms of edges and nodes. When compared to 100 random networks, each network except the aboveground organic and belowground conventional networks consistently had a significantly (*p* < 0.05) different degree distribution than 100 random networks (Table 3). Since the aboveground organic network and belowground conventional network did not have a significantly different degree distribution than a random network, they will not be interpreted further. All networks contained a greater number of prokaryotic than fungal nodes and this difference was more pronounced belowground. Overall, networks had a diverse mix of bacterial and fungal phyla but were dominated by Proteobacteria and Actinobacteria with fungal nodes primarily being Ascomycota (Supplementary Figure S8).

**TABLE 3:** Summary table of network statistics displaying: number of nodes, number of edges, network stability, network sparsity, modularity, number of modules, number of fungal nodes, number of prokaryotic nodes, and number of detected hub species in above and belowground networks of conventional, no-till, and organic management systems.

**Network**	**# of nodes**	**# of edges**	**stability**	**sparsity**	**modularity**	**# of modules**	**# Fungal nodes**	**# Prokaryotic nodes**	**# of hubs**	***P*-value range**
Belowground conventional*	139	270	0.047	0.0279	0.44	38	26	113	3	0.016–0.27
Aboveground conventional	96	173	0.0495	0.038	0.61	20	42	54	2	**2.3E-04–0.037**
Belowground no-till	441	2663	0.0495	0.0274	0.36	8	36	415	5	**1.3E-03–0.027**
Aboveground no-till	119	270	0.0493	0.0381	0.53	16	46	73	2	**2.2E-05–0.031**
Belowground organic	424	2232	0.0487	0.0248	0.34	22	26	398	1	**3.9E-05–0.037**
Aboveground organic*	52	51	0.046	0.0377	0.7	17	16	36	0	8.5E-03–0.57

P-values represent the range of p-values calculated when comparing the network to 100 random networks. Significant values (P < 0.05) are shown in bold. Networks with (*) denote those that were not consistently different from random networks.

**FIGURE 7:**
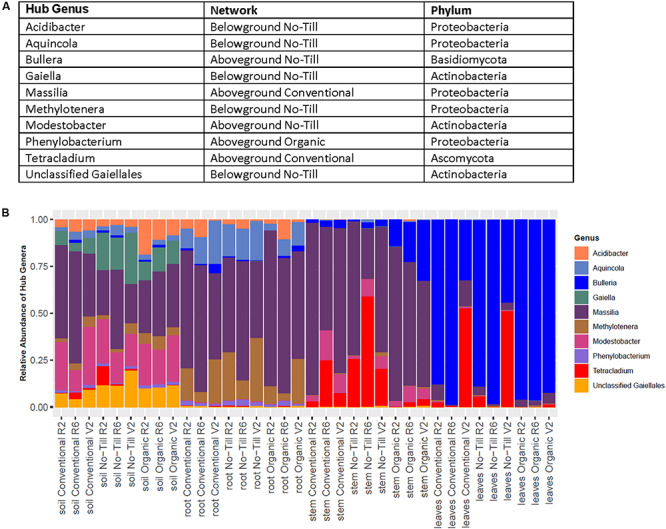
Summary of hub taxa detected in above and belowground bipartite networks for conventional, no-till, and organic management systems displayed as **(A)** a table of detected hub genera and **(B)** stacked barplot showing the distributions of hub taxa across all managements and sample origins.

Within above and belowground networks created for each management system, 10 hubs were identified from significant networks to belong to 10 separate fungal and bacterial genera (Figure 7A). Most bacterial hubs consisted of Proteobacteria and Actinobacteria while the two fungal hubs were one basidiomycete and one ascomycete. The hub OTUs varied in relative abundance, the *Massilia* OTU 17 and *Bulleria* OTU 10 were dominant among hubs in the roots, stems, and leaves (Figure 7B). Some hubs varied in relative abundance by management system, for example, *Tetracladium* OTU 59 was less relatively abundant in organic leaves and stems compared to no-till and conventional samples. Most hub OTUs were restricted to one compartment or to only above or belowground samples. This was not the case for *Massilia* OTU 17 which was present throughout and *Modestobacter* OTU 116 which was present in the soil and stems (Figure 7B).

## Discussion

In this study, we assessed impacts of long-term cropping management systems on the soybean microbiome at a unique agricultural LTER site with 30 years of consistent management. We detected differences in the soybean-associated microbiome between management systems and growth stages throughout all sample origins. It is important to note that since plant compartments are not independent of each other; detected differences between managements in non-soil compartments may be largely driven by differences in the soil due to the role of the soil in seeding the microbiome of plant compartments (Grady et al., 2019). However, if this is the case, our results demonstrate that differences from the soil persist throughout the plant. Additionally, since the same plots were sampled repeatedly throughout the season, samples from the same plot at different time points are not completely independent. However, when differences by growth stage are the highest (in the leaves and stems) the samples cluster by growth stage even when they are from different management systems; indicating that this is likely a true effect of growth stage, not simply differences between plots that persist due to repeated sampling of that plot (Supplementary Figure S2). Some effect of growth stage may in fact be obscured due to differences between plots that persist because of repeated sampling of the same plots. Future studies performed at multiple sites can identify taxa which are consistently affected by the growth stage of the host plant at multiple sites.

In terms of alpha diversity, there was not a consistent difference between organic and conventional management systems, a pattern that was also observed for corn (Chen H. et al., 2018). Alpha diversity results were consistent with studies that have demonstrated the highest alpha diversity of both Fungi and Prokaryotes in the soil (Lebreton et al., 2019; Suárez-Moo et al., 2019). Additionally our results were consistent with previous results from the same site which demonstrated that the highest within plant alpha diversity for Prokaryotes could be found in the roots, but for Fungi the root alpha diversity was similar to that of the leaves (Gdanetz and Trail, 2017). Interestingly, previous studies have demonstrated higher alpha diversity of fungal communities in *Populus* stems compared to leaves which contradicts our results (Cregger et al., 2018). This may be due to differences in plants, or the level at which stems were sampled. Within a single compartment, in terms of alpha diversity, the primary pattern in fungal and prokaryotic communities was a decrease in richness in the early reproductive (R2) stage followed by an increase at the late reproductive (R6) growth stage. This differed from a trend of increasing alpha diversity in plant organs throughout the season, as was detected in a previous study on wheat at the KBS LTER (Gdanetz and Trail, 2017). Our results also differed from a previous observation of a reduction in phyllosphere prokaryotic diversity throughout a soybean growing season (Copeland et al., 2014). Additionally, fungal richness was lower in organic stems and leaves. It is possible that this was due to management but it could also be due to the different plant genotype that was used in the organic system, as has been demonstrated to be an important source of variation in the maize rhizosphere (Peiffer et al., 2013). Taken together, these observations suggest that trends in alpha diversity are not consistent across crops and sites. This may indicate that alpha diversity and other microbial community measures may be altered by unmeasured environmental factors as well as biotic factors such as plant exudates, interspecies competition, and the effects of non-microbial taxa (Jones et al., 2019).

The structures of the fungal communities were distinct between management regimes in terms of the presence and absence of particular fungal genera. For example, although abundant in other treatments, in no-till soils, *Podospora* and *Didymyella* were below the 4% threshold to be included in bar graphs (Figure 1A). *Podospora* has been identified previously as being more abundant in conventionally tilled wheat soils (Degrune et al., 2017). In the soil, it is postulated that tillage can alter fungal communities such as arbuscular mycorrhizal fungi (AMF) by disrupting hyphae (Sharma-Poudyal et al., 2017). Consistent with this hypothesis, the highest relative abundance of AMF was detected in no-till soils, but mechanistic studies are needed to ensure that this difference is due to tillage at the KBS LTER site.

Indicator species analysis identified taxa such as *Mortierella* and *Minimedusa* that were associated with organic and conventional management systems (Figure 6A). These same taxa were identified as being important in assigning samples to management systems in random forest models (Supplementary Figure S4). *Minimedusa polyspora* is of interest because it has been suggested to be plant growth promoting given its ability to solubilize phosphorous (Ceci et al., 2018). Some *Mortierella* species are also known to solubilize phosphorus (Osorio and Habte, 2013). *Mortierella elongata* has been reported to upregulate nutrient uptake and lipid signaling pathways in *Populus* (Liao et al., 2019), and are known to break down toxic organic compounds in the soil (Li et al., 2018).

*Phoma* was enriched in aboveground stem and leaf fungal communities in organic managements, while, *Fusarium* and *Phaeosphaeriopsis* were conspicuously absent (Figures 1C,D). Additionally, various *Phoma* OTUs were identified as being important for separating belowground management systems in random forest models (Supplementary Figure S4A). Interestingly, *Phoma* spp. have been indicated as a possible biocontrol agent for *Fusarium graminearum* in wheat, which may explain the lack of *Fusarium* where *Phoma* was abundant (Gdanetz and Trail, 2017). Indicator species analysis identified *Fusarium* sp. as statistically associated to aboveground soybean tissues in conventional and no-till managements. This result was interesting because previous work at the same site found *Fusarium* to be enriched in the phyllosphere of organic wheat (Gdanetz and Trail, 2017). It is also possible that this microbiome difference is due to the difference in soybean cultivar used in the organic system, as host genotype differences have been demonstrated in grape, maize, and poplar phyllospheres (Bálint et al., 2013; Mezzasalma et al., 2018; Chen et al., 2019).

In soil prokaryotic communities, Spartobacteria were enriched in no-till treatments. Spartobacteria has been found to be associated with no -till corn/soybean fields in a previous study, indicating that tillage regime may be specifically disruptive to these bacteria (Smith et al., 2016). The no-till prokaryotic community was enriched in *Bradyrhizobium.* Previous studies have found a positive correlation between *Bradyrhizobium* and increased organic carbon caused by not tilling (Yan et al., 2014). No-till and organic management regimes have been demonstrated to significantly increase total carbon in surface soils at the KBS LTER, which may explain the enrichment of *Bradyrhizobium* in the no-till management system (Syswerda et al., 2011). However, since soil carbon was not measured as a part of this study, further work is needed to establish this relationship. In aboveground tissues, *Hymenobacter* was enriched in the no-till and conventional management systems. Some *Hymenobacter* species are plant growth promoting bacteria that can increase fatty acid content of plants (Dimitrijević et al., 2017; Aydogan et al., 2018). Together, these results indicate that management choices may select for beneficial microbes, but strain level identifications of taxa will be needed to assess this hypothesis. The indicator species analysis identified taxa which were tightly associated with roots or soils or tightly associated with specific growth stages (Figure 6C). For example, *Aureimonas* appeared only in the early reproductive (R2) and late reproductive (R6) growth stage of the three management systems. This observation is consistent with the idea that plants can recruit diverse microbes throughout their life cycles as they develop and their environment changes (Rodriguez et al., 2019). It is important to note that future studies on the effect of management regimes on the soybean microbiome are unlikely to identify the exact same indicator taxa. However, future work and more mechanistic studies may identify classes of microbes likely to be highly impacted by agricultural management. This information could then be used to predict the effect of the microbiome on plant health under alternative agricultural management.

The main explanatory variable of beta diversity in the soybean microbiome appeared to be whether the sample was from above or below ground compartments (Figures 5A,F). This result agrees with previous microbiome studies in *Arabidopsis* and wheat which showed different microbial communities are present in above and belowground plant tissues (Gdanetz and Trail, 2017; Bergelson et al., 2019). At a finer resolution, there was separate clustering for leaves and stems and roots and soils, as has been noted in prokaryotic and fungal communities in the *Populus* microbiome (Cregger et al., 2018). Differences between microbial communities of organic vs. non-organic management systems have been demonstrated in grape and apple (Ottesen et al., 2009; Schmid et al., 2011). Alternatively, pronounced effects of plant genotype could be driving differences in the phyllosphere fungal community, as has been reported for *Populus* (Bálint et al., 2013; Cregger et al., 2018). However, there were also distinct fungal communities between conventional and no-till management systems that persisted throughout the growing season in various plant compartments (Supplementary Table S4A and Supplementary Figure S5). Differences between conventional and no-till management systems were also made clear by the low error rate of random forest analyses in distinguishing agricultural management regimes (Supplementary Figures S4A,B). Tillage is known to be damaging to fungal mycelial networks in the soil, reducing the ratio of fungal to bacterial cells in soils (Beare et al., 1997). Consequently, changes in fungal communities were expected given the substantial differences between tilled and non-tilled soils as has been demonstrated previously (Sharma-Poudyal et al., 2017). Differences between conventional and no-till management systems were not only in the soil but persisted in the leaves throughout the growing season (Supplementary Figure 5G). The effect of no-till vs. conventional agricultural management on the fungal communities of aboveground plant compartments has been understudied but may have an important impact on plant health. Our study found shifts in the phyllosphere community throughout a growing season, and is consistent with previous observations of seasonal phyllosphere shifts in fungal and bacterial communities at the KBS LTER in wheat, switchgrass and miscanthus (Gdanetz and Trail, 2017; Grady et al., 2019). While PERMANOVA results confirm the effect of sampling time-point on aboveground plant microbiome compartments, they also confirm the effects of crop management regime on soil and rhizobiome (Tables 1, 2). Further work is warranted in this area to determine if time- point shifts are driven by deterministic or stochastic effects.

PCoA plots of prokaryotic communities did not show a clear signature of management system on the soybean microbiome (Figures 5G–J), yet a clustering of growth stages is evident in aboveground tissues (Supplementary Figure S2). PERMANOVA results showed that management system played a larger role in the soil and growth stage/sampling point played a larger role in plant tissues, but the effects of both factors were significant in all sample origins (Tables 1, 2). The moderate but significant effect of management regime on soil prokaryotic communities was consistent with results of a previous study that compared organic and conventional management systems (Chen H. et al., 2018). As with fungal communities, changes in aboveground and root prokaryotic communities based on plant growth stage and sampling time-point are consistent with the results of previous studies on maize and rice (Manching et al., 2017; Edwards et al., 2018). Differences in assembly between above and belowground tissues may alter the community’s ability to respond to agricultural management and plant growth. Similar to Fungi, when the organic management system was not included in analyses, there was still a significant difference between conventional and no-till management systems, although the difference was smaller than in fungal communities (Supplementary Table S4B and Supplementary Figure S7). As with Fungi, this distinction between conventional and no-till agriculture has been demonstrated in soils, but has been understudied within plant compartments (Piazza et al., 2019). Additionally, in the leaves the effect of management regime was reduced throughout the growing season when analyzing no-till and conventionally managed treatments alone (Supplementary Figure S7G and Supplementary Table S4B).

Microbial networks in the long-term no-till management were denser than those of conventional or organic managements. This is undoubtedly related to higher prokaryotic alpha diversity in the no-till management system. We speculate that the increased number of core taxa, and therefore nodes, in the no-till networks may be related to both the lack of disturbance and increased soil carbon quality and quantity associated with no-till (Smith et al., 2016; Banerjee et al., 2019). Differences in network density and other network statistics between organic and other management systems may be due to management regime or due to host genotype differences. Further mechanistic studies are needed to assess the effects of more complex networks on host plant health.

Microbial networks detected different hub species in each network. Due to the lack of taxonomic resolution in amplicon sequencing studies, species and strain level identification of hubs is impossible. However, detection of hub OTUs belonging to particular microbial genera may inform future mechanistic studies. In the no-till belowground network, two detected hubs were from the Gaillales order, which has been previously shown to be enriched in the roots of rice compared to surrounding soil, their detection as hubs may indicate an important role in structuring the root microbiome (Hernández et al., 2015). The only hub OTU detected in the belowground organic network was *Phenylobacterium*. This particular OTU seemed to only appear at a low relative abundance among hubs and only appeared in the roots and soil. Species from this genus have been understudied in terms of their effect on plant health, but it’s detection as a hub in the roots indicates that it may play a role in structuring the root microbiome.

One hub of the aboveground no-till network was a fungus belonging to the genus, *Bullera*. Similar to many other basidiomycete yeasts, *Bullera* species have been isolated from the phyllosphere of various plants, but their roles in plant health are undetermined (Wang et al., 2016). The only other fungal hub detected was a *Tetracladium* OTU which was a hub in the aboveground conventional network and was previously found to be abundant at the KBS-LTER site (Gdanetz and Trail, 2017). *Massilia* is another aboveground hub taxon of interest. Although studied primarily in the roots, taxa from this genus are potentially beneficial due to their ability to solubilize phosphate (Silva et al., 2017). Its presence in aboveground tissues indicates that it may be important in structuring plant microbiomes in both above and belowground phytobiomes. Further research is needed to determine why hub taxa are highly connected to other microbial members and how these connections help assemble soybean-associated microbial communities.

## Conclusion

Here we report on the impact of long-term cropping management systems on the soybean microbiome. In doing so, we also addressed whole plant-microbiome changes in above and belowground compartments across the growing season. Our results indicate that the management system and growth stage have significant effects on the soybean microbiome. The effect of management system persisted when comparing conventional and no-till systems, excluding organic samples that were of a different genotype. Our results also indicated that specific indicator taxa varied between management regimes. Some of the indicator taxa such as *Mortierella* and *Hymenobacter* may be beneficial to plants. Additionally, the management system altered the network hub taxa, which may be important in structuring the microbiome. Some hub OTUs, such as *Massilia*, belonged to microbial genera that are known to contain plant beneficial organisms. Taken together, these results indicate that agricultural management practices impact whole-plant microbiomes. How specific management regimes can be employed to select desired microbial traits is still an open question. Further research into taxa identified by indicator species and network analyses may help to elucidate their functional roles to explain why specific taxa may be enriched under different management systems.

## Data Availability Statement

The datasets generated for this study can be found in the NCBI SRA Archive: PRJNA603147, PRJNA603199, and PRJNA603207.

## Author Contributions

GB, FT, and MC designed experiments and secured funding. RL, ZN, FT, MC, and GB collected and processed samples. RL, GMB, and ZN analyzed data and created figures. RL, GMB, ZN, and GB wrote the manuscript. All authors approved the final manuscript draft.

## Conflict of Interest

The authors declare that the research was conducted in the absence of any commercial or financial relationships that could be construed as a potential conflict of interest.

## Footnotes

1 http://www.bioinformatics.babraham.ac.uk/projects/fastqc/
