# Supplementary material for: Crop Management Impacts the Soybean (Glycine max) Microbiome
Source: Front Microbiol. 2020 Jun 3;11:1116. doi: 10.3389/fmicb.2020.01116 (PMC7283522; doi:10.3389/fmicb.2020.01116)
Supplement: Supplementary file 1 [file Data_Sheet_1.docx]

Supplementary Material

**Supplementary Fig 1** Rarefaction curves representing the number of OTUs detected per number of reads produced in sequencing of  fungal communities **(A)** associated with soil samples, **(B)** associated with soybean root samples, **(C)** associated with soybean stem samples, **(D)** associated with soybean leaf samples and prokaryotic communities (**E)** associated with soil samples, **(F)** associated with soybean root samples, **(G)** associated with soybean stem samples, and **(H)** associated with soybean leaf samples.

**Supplementary Fig 2** Principal coordinates analysis plots, based on Bray-Curtis dissimilarity, of fungal **(A)** communities associated with soybean soil, root, stem, and leaf samples, **(B)** associated with  soil samples, **(C)** associated with root samples **(D)**associated with stem samples, **(E)** associated with leaf samples and prokaryotic **(F)** communities associated with soil, root, stem, and leaf samples, **(G)** associated with soil samples, **(H)** associated with root samples, **(I)** associated with stem samples, and **(J)** associated with leaf samples. The shape represents the plant growth stage, while color represents sample origin in **(A)** and **(F)**.  In all others the color represents the plant growth stage.

**Supplementary Fig 3** Principal coordinates analysis plots using Bray-Curtis dissimilarity of fungal **(A)** communities associated with soybean soil split by soybean growth stage, **(B)** communities associated with soybean soil split by agricultural management system, **(C)** communities associated with soybean roots split by soybean growth stage, **(D)** communities associated with soybean roots split by agricultural management system, **(E)** communities associated with soybean stems split by soybean growth stage, **(F)** communities associated with soybean stems split by agricultural management system, **(G)** communities associated with soybean leaves split by soybean growth stage, and **(H)** communities associated with soybean leaves split by agricultural management system.

**Supplementary Fig 4** Random forest modelling results of fungal communities **(A)** associated with belowground samples, **(B)** communities associated with aboveground plant tissues, and prokaryotic communities **(C)** associated with belowground samples, and **(D)** communities associated with aboveground plant tissues.

**Supplementary Fig 5** Principal coordinates analysis plots using Bray-Curtis dissimilarity, excluding the organic management of fungal **(A)** communities associated with soybean soil split by soybean growth stage, **(B)** communities associated with soybean soil split by agricultural management system, **(C)** communities associated with soybean roots split by soybean growth stage, **(D)** communities associated with soybean roots split by agricultural management system, **(E)** communities associated with soybean stems split by soybean growth stage, **(F)** communities associated with soybean stems split by agricultural management system, **(G)** communities associated with soybean leaves split by soybean growth stage, and **(H)** communities associated with soybean leaves split by agricultural management system.

**Supplementary Figure 6** Principal coordinates analysis plots using Bray-Curtis dissimilarity,  of prokaryotic communities **(A)** associated with soybean soil split by soybean growth stage, **(B)** associated with soybean soil split by agricultural management system, **(C)** associated with soybean roots split by soybean growth stage, **(D)** associated with soybean roots split by agricultural management system, **(E)** \associated with soybean stems split by soybean growth stage, **(F)** associated with soybean stems split by agricultural management system, **(G)** associated with soybean leaves split by soybean growth stage, and those **(H)** associated with soybean leaves split by agricultural management system.

**Supplementary Figure 7** Principal coordinates analysis plots using Bray-Curtis dissimilarity, excluding the organic management of prokaryotic communities **(A)** associated with soybean soil split by soybean growth stage, **(B)** associated with soybean soil split by agricultural management system, **(C)** associated with soybean roots split by soybean growth stage, **(D)** associated with soybean roots split by agricultural management system, **(E)** associated with soybean stems split by soybean growth stage, **(F)** associated with soybean stems split by agricultural management system, **(G)** associated with soybean leaves split by soybean growth stage, and **(H)** associated with soybean leaves split by agricultural management system.


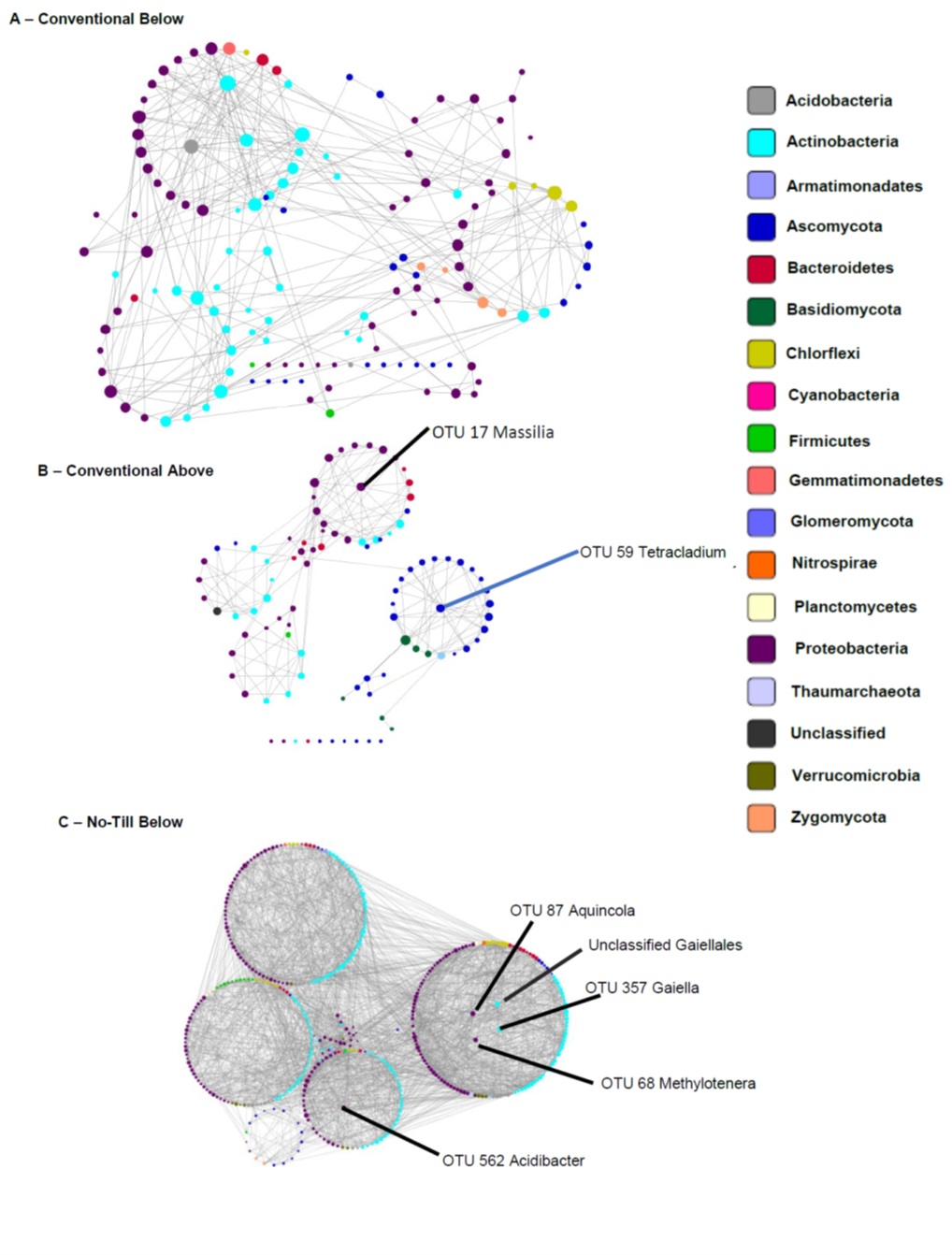

**Supplementary Figure 8** Bipartite networks constructed for fungal and prokaryotic communities of **(A)** belowground conventional samples, **(B)** aboveground conventional samples, **(C)** belowground no-till samples, **(D)** aboveground no-till samples, **(E)** belowground organic samples, and **(F)** aboveground organic samples.

| **Fungi and Soil Prokaryotes Cycling** | | | | | | | |  |
| --- | --- | --- | --- | --- | --- | --- | --- | --- |
| **Step 1** |  |  | **Step 2** |  |  | **Step 3** |  |  |
| **Time** | **Temperature (c)** | **Cycles** | **Time** | **Temperature (c)** | **Cycles** | **Time** | **Temperature (c)** | **Cycles** |
| 5:00 | 95 |  | 5:00 | 95 |  | 5:00 | 95 |  |
| 0:30 | 95 | 10X | 0:35 | 95 | 10X | 0:40 | 95 | 15X |
| 0:30 | 50 |  | 0:35 | 50 |  | 0:50 | 63 |  |
| 1:00 | 72 |  | 1:10 | 72 |  | 1:20 | 72 |  |
| 7:00 | 72 |  | 7:00 | 72 |  | 7:00 | 72 |  |
| Infinite | 10 |  | Infinite | 10 |  | Infinite | 10 |  |
|  |  |  |  |  |  |  |  |  |
| **Plant Tissue Prokaryotes Cycling** | | | | | | | |  |
| **Step 1** |  |  | **Step 2** |  |  | **Step 3** |  |  |
| **Time** | **Temperature (c)** | **Cycles** | **Time** | **Temperature (c)** | **Cycles** | **Time** | **Temperature (c)** | **Cycles** |
| 5:00 | 95 |  | 5:00 | 95 |  | 5:00 | 95 |  |
| 0:30 | 95 | 10X | 0:30 | 95 | 10X | 0:40 | 95 | 15X |
| 0:15 | 75 |  | 0:15 | 75 |  |  |  |  |
| 0:30 | 50 |  | 0:35 | 50 |  | 0:50 | 63 |  |
| 0:45 | 72 |  | 0:50 | 72 |  | 1:20 | 72 |  |
| 7:00 | 72 |  | 7:00 | 72 |  | 7:00 | 72 |  |
| Infinite | 10 |  | Infinite | 10 |  | Infinite | 10 |  |

**Supplementary Table 1.** Thermocycling conditions used for amplifying fungal (ITS) and bacterial (16S) genes from all sample origins.

| **Plant Associated Prokaryotes** | | **Fungi** | |
| --- | --- | --- | --- |
| **Step 1** | | **Step 1** | |
| **Component** | **Volume/Sample (uL)** | **Component** | **Volume/Sample (uL)** |
| 2X Platinum Green Taq Master Mix(Thermo Fisher,USA) | 6.25 | 2X Dream Taq Green PCR Master Mix (Thermo Fisher) | 6.25 |
| 10 uM 515F Primer (IDT, USA) | 0.375 | 10 uM ITS 1F Primer (IDT,USA) | 0.375 |
| 10 uM 806R Primer (IDT, USA) | 0.375 | 10 uM ITS 4 Primer (IDT,USA) | 0.375 |
| Bovine Serum Albumin (BSA, 3%) | 1 | Bovine Serum Albumin (BSA, 3%) | 1 |
| 50 uM Mitochondrial PNA clamp (PNA Bio, USA) | 0.18 | H2O | 3 |
| 50 uM Plastid PNA clamp (PNA Bio, USA) | 0.18 | Extracted DNA | 1 |
| GC Enhancer (Thermo Fisher,USA) | 2 | **Step 2** | |
| H2O | 0.64 | **Component** | **Volume/sample (uL)** |
| Extracted DNA | 1 | 2X Dream Taq Green PCR Master Mix (Thermo Fisher) | 6.25 |
| **Step 2** | | 10 uM ITS 1F Primer Frameshift (IDT,USA) | 0.375 |
| **Component** | **Volume/Sample (uL)** | 10 uM ITS 4 Primer Frameshift (IDT,USA) | 0.375 |
| 2X Platinum Green Taq Master Mix(Thermo Fisher,USA) | 6.25 | Bovine Serum Albumin (BSA, 3%) | 1 |
| 10 uM 515F Primer Frameshift (IDT, USA) | 0.375 | H2O | 2 |
| 10 uM 806R Primer Frameshift (IDT, USA) | 0.375 | Step 1 Product | 2 |
| Bovine Serum Albumin (BSA, 3%) | 0.64 | **Step 3** | |
| 50 uM Mitochondrial PNA clamp (PNA Bio, USA) | 0.18 | **Component** | **Volume/sample (uL)** |
| 50 uM Plastid PNA clamp (PNA Bio, USA) | 0.18 | 2X Dream Taq Green PCR Master Mix (Thermo Fisher) | 8 |
| GC Enhancer (Thermo Fisher,USA) | 2 | Barcode Forward Primer | 0.5 |
| Step 1 Product | 2 | Bovine Serum Albumin (BSA, 3%) | 1 |
| **Step 3** | | H2O | 0.5 |
| **Component** | **Volume/Sample (uL)** | Unique 10 Nucleotide Barcode | 1 |
| 2X Platinum Green Taq Master Mix (Thermo Fisher,USA) | 8 | Step 2 Product | 4 |
| Barcode Forward Primer | 0.5 |  |  |
| Bovine Serum Albumin (BSA, 3%) | 1 | **Soil Prokaryote PCR Mixes are the same as Fungi, but with Platinum Taq** | |
| GC Enhancer (Thermo Fisher,USA) | 0.5 |  |  |
| Unique 10 Nucleotide Barcode | 1 |  |  |
| Step 2 Product | 4 |  |  |

**Supplementary Table 2.** PCR recipes for amplifying the ITS and 16S markers.

|  | **Factor** | **PERMANOVA** | | | |  | **DISPERSION** | |
| --- | --- | --- | --- | --- | --- | --- | --- | --- |
|  | **A - Fungi** | **Df** | **F-value** | **R2** | **P-value** |  | **F-value** | **P-value** |
| **Soil** | Growth Stage | 2 | 4.347 | 0.071 | **0.0001** |  | 2.566 | 0.08580 |
|  | Management | 2 | 9.794 | 0.160 | **0.0001** |  | 8.123 | **0.00056** |
|  | Growth Stage:Management | 4 | 1.948 | 0.064 | **0.0001** |  |  |  |
|  | Residuals | 86 |  |  |  |  |  |  |
|  | Total | 94 |  |  |  |  |  |  |
| **Roots** |  | **Df** | **F-value** | **R2** | **P-value** |  | **F-value** | **P-value** |
|  | Growth Stage | 2 | 4.310 | 0.075 | **0.0001** |  | 3.525 | **0.03337** |
|  | Management | 2 | 4.198 | 0.073 | **0.0001** |  | 0.709 | 0.49480 |
|  | Growth Stage:Management | 4 | 2.286 | 0.079 | **0.0001** |  |  |  |
|  | Residuals | 89 |  |  |  |  |  |  |
|  | Total | 97 |  |  |  |  |  |  |
| **Stems** |  | **Df** | **F-value** | **R2** | **P-value** |  | **F-value** | **P-value** |
|  | Growth Stage | 2 | 8.324 | 0.138 | **0.0001** |  | 0.279 | 0.7571 |
|  | Management | 2 | 8.699 | 0.144 | **0.0001** |  | 4.553 | **0.0132** |
|  | Growth Stage:Management | 4 | 1.891 | 0.063 | **0.002** |  |  |  |
|  | Residuals | 79 |  |  |  |  |  |  |
|  | Total | 87 |  |  |  |  |  |  |
| **Leaves** |  | **Df** | **F-value** | **R2** | **P-value** |  | **F-value** | **P-value** |
|  | Growth Stage | 2 | 59.172 | 0.433 | **0.0001** |  | 7.763 | **7.24E-04** |
|  | Management | 2 | 15.091 | 0.110 | **0.0001** |  | 0.032 | 0.9685 |
|  | Growth Stage:Management | 4 | 6.998 | 0.102 | **0.0001** |  |  |  |
|  | Residuals | 97 |  |  |  |  |  |  |
|  | Total | 105 |  |  |  |  |  |  |
|  | **B-Prokaryotes** | **Df** | **F-value** | **R2** | **P-value** |  | **F-value** | **P-value** |
| **Soil** | Growth Stage | 2 | 3.829 | 0.063 | **0.0001** |  | 5.086 | **0.0079** |
|  | Management | 2 | 7.526 | 0.119 | **0.0001** |  | 8.555 | **0.0004** |
|  | Growth Stage:Management | 4 | 1.882 | 0.060 | **0.0002** |  |  |  |
|  | Residuals | 92 |  |  |  |  |  |  |
|  | Total | 101 |  |  |  |  |  |  |
| **Roots** |  | **Df** | **F-value** | **R2** | **P-value** |  | **F-value** | **P-value** |
|  | Growth Stage | 2 | 7.087 | 0.110 | **0.0001** |  | 3.477 | **0.0350** |
|  | Management | 2 | 4.405 | 0.069 | **0.0001** |  | 3.055 | 0.0514 |
|  | Growth Stage:Management | 4 | 2.149 | 0.067 | **0.0007** |  |  |  |
|  | Residuals | 97 |  |  |  |  |  |  |
|  | Total | 105 |  |  |  |  |  |  |
| **Stems** |  | **Df** | **F-value** | **R2** | **P-value** |  | **F-value** | **P-value** |
|  | Growth Stage | 2 | 19.511 | 0.253 | **0.0001** |  | 8.146 | **8.96E-05** |
|  | Management | 2 | 8.932 | 0.116 | **0.0001** |  | 10.287 | **0.0001** |
|  | Growth Stage:Management | 4 | 1.784 | 0.046 | **0.0205** |  |  |  |
|  | Residuals | 90 |  |  |  |  |  |  |
|  | Total | 98 |  |  |  |  |  |  |
| **Leaves** |  | **Df** | **F-value** | **R2** | **P-value** |  | **F-value** | **P-value** |
|  | Growth Stage | 2 | 33.654 | 0.366 | **0.0001** |  | 29.286 | **1.24E-10** |
|  | Management | 2 | 4.557 | 0.050 | **0.0001** |  | 1.431 | 0.2442 |
|  | Growth Stage:Management | 4 | 4.559 | 0.099 | **0.0001** |  |  |  |
|  | Residuals | 89 |  |  |  |  |  |  |
|  | Total | 97 |  |  |  |  |  |  |

**Supplementary Table 3:** Permutational multivariate analysis of variance (*adonis*) and multivariate homogeneity of groups dispersions analysis (*betadisper*) results for (**A)** fungal communities associated with soybean soil, root, stem, and leaf samples, **(B)** prokaryotic communities associated with soybean soil, root, stem, and leaf samples. Significance values at p ≤ .05 are indicated in bold.

| **A - Fungi** | | **PERMANOVA** | | |  | **DISPERSION** | |
| --- | --- | --- | --- | --- | --- | --- | --- |
| **Growth Stage** | | **F-value** | **R2** | **P-value** |  | **F-value** | **P-value** |
| **Soil** | V2 | 4.515 | 0.17 | **1.00E-04** |  | 0.043 | 0.8377 |
|  | R2 | 3.871 | 0.17 | **1.00E-04** |  | 0.4581 | 0.507 |
|  | R6 | 4.241 | 0.17 | **1.00E-04** |  | 0.4612 | 0.505 |
| **Roots** | V2 | 4.05 | 0.16 | **3.00E-04** |  | 0.831 | 0.372 |
|  | R2 | 2.16 | 0.102 | **1.60E-02** |  | 4.83 | **0.041** |
|  | R6 | 2.06 | 0.089 | **7.60E-03** |  | 0.778 | 0.387 |
| **Stems** | V2 | 3.96 | 0.16 | **1.00E-04** |  | 0.339 | 0.567 |
|  | R2 | 2.33 | 0.11 | **9.40E-03** |  | 2.723 | 0.115 |
|  | R6 | 7.5 | 0.283 | **1.00E-04** |  | 2.394 | 0.138 |
| **Leaves** | V2 | 8.62 | 0.29 | **1.00E-04** |  | 1.867 | 0.186 |
|  | R2 | 3.57 | 0.15 | **8.00E-04** |  | 2.645 | 0.119 |
|  | R6 | 7.27 | 0.25 | **1.00E-04** |  | 0.169 | 0.291 |
| **Management** | | **F-value** | **R2** | **P-value** |  | **F-value** | **P-value** |
| **Soil** | Conventional | 1.57 | 0.095 | **1.00E-03** |  | 0.453 | 0.639 |
|  | No-Till | 2.22 | 0.125 | **1.00E-04** |  | 0.825 | 0.448 |
| **Roots** | Conventional | 2.79 | 0.161 | **3.00E-04** |  | 0.209 | 0.812 |
|  | No-Till | 4.42 | 0.216 | **1.00E-04** |  | 4.712 | **0.016** |
| **Stems** | Conventional | 3.18 | 0.175 | **1.00E-04** |  | 0.997 | 0.381 |
|  | No-Till | 4.69 | 0.244 | **1.00E-04** |  | 0.462 | 0.634 |
| **Leaves** | Conventional | 27.35 | 0.638 | **1.00E-04** |  | 2.39 | 0.108 |
|  | No-Till | 29.53 | 0.642 | **1.00E-04** |  | 1.21 | 0.31 |
| **B - Prokaryotes** | | **PERMANOVA** | | |  | **DISPERSION** | |
| **Growth Stage** | | **F-value** | **R2** | **P-value** |  | **F-value** | **P-value** |
| **Soil** | V2 | 6.504 | 0.228 | **1.00E-04** |  | 1.656 | 0.212 |
|  | R2 | 3.124 | 0.135 | **6.00E-04** |  | 0.951 | 0.341 |
|  | R6 | 3.301 | 0.13 | **1.00E-04** |  | 16.913 | **4.58E-04** |
| **Roots** | V2 | 2.731 | 0.115 | **1.69E-02** |  | 2.98 | 0.099 |
|  | R2 | 0.694 | 0.032 | 7.68E-01 |  | 0.0309 | 0.862 |
|  | R6 | 2.213 | 0.0914 | **2.71E-02** |  | 1.523 | 0.23 |
| **Stems** | V2 | 2.864 | 0.12 | **5.30E-03** |  | 1.127 | 0.3 |
|  | R2 | 3.679 | 0.143 | **6.00E-04** |  | 0.347 | 0.5621 |
|  | R6 | 3.567 | 0.151 | **1.00E-04** |  | 1.392 | 0.251 |
| **Leaves** | V2 | 5.108 | 0.203 | **1.00E-04** |  | 1.709 | 0.206 |
|  | R2 | 1.418 | 0.0662 | 2.15E-01 |  | 2.492 | 0.13 |
|  | R6 | 2.406 | 0.0986 | **1.21E-02** |  | 0.869 | 0.361 |
| **Management** | | **F-value** | **R2** | **P-value** |  | **F-value** | **P-value** |
| **Soil** | Conventional | 2.972 | 0.157 | **1.00E-04** |  | 29.747 | **5.00E-08** |
|  | No-Till | 2.259 | 0.124 | **1.30E-03** |  | 1.023 | 0.371 |
| **Roots** | Conventional | 2.359 | 0.132 | **7.80E-03** |  | 0.165 | 0.849 |
|  | No-Till | 3.494 | 0.175 | **6.00E-04** |  | 2.87 | 0.071 |
| **Stems** | Conventional | 5.33 | 0.262 | **1.00E-04** |  | 1.19 | 0.319 |
|  | No-Till | 10.06 | 0.378 | **1.00E-04** |  | 0.627 | 0.541 |
| **Leaves** | Conventional | 15.56 | 0.509 | **1.00E-04** |  | 8.53 | **1.20E-03** |
|  | No-Till | 16.79 | 0.512 | **1.00E-04** |  | 5.38 | **0.0097** |

**Supplementary Table 4:**  Permutational multivariate analysis of variance (*adonis*) and multivariate homogeneity of groups dispersions analysis (*betadisper*) results for **(A)** fungal communities associated with soybean soil, root, stem, and leaf samples showing the effect of agricultural management on individual growth stages and the effect of growth stage on individual agricultural management systems, and (**B)** prokaryotic communities associated with soybean soil, root, stem, and leaf samples showing the effect of agricultural management on individual growth stages and the effect of growth stage on individual agricultural management systems.
